# Prevalence and Correlates of Lymphatic Filariasis Infection and Its Morbidity Following Mass Ivermectin and Albendazole Administration in Mkinga District, North-Eastern Tanzania

**DOI:** 10.3390/jcm9051550

**Published:** 2020-05-21

**Authors:** Adam M. Fimbo, Omary M.S. Minzi, Bruno P. Mmbando, Abbie Barry, Alex F. Nkayamba, Kissa W. Mwamwitwa, Alpha Malishee, Misago D. Seth, Williams H. Makunde, Parthasarathi Gurumurthy, John P.A. Lusingu, Appolinary A.R. Kamuhabwa, Eleni Aklillu

**Affiliations:** 1 Division of Clinical Pharmacology, Department of Laboratory Medicine, Karolinska Institutet at Karolinska University Hospital, 141 86 Huddinge, Sweden; adamfimbo@gmail.com (A.M.F.); abbie.barry@ki.se (A.B.); 2 Tanzania Medicines and Medical Devices Authority (TMDA), Dar es Salaam, P.O. Box 77150, Tanzania; alexnkayamba@yahoo.com (A.F.N.); ki313ssa@yahoo.com (K.W.M.); 3 Department of Clinical Pharmacy and Pharmacology, School of Pharmacy, Muhimbili University of Health and Allied Sciences, Dar es Salaam, P.O. Box 65001, Tanzania; minziobejayesu@gmail.com (O.M.S.M.); enali2012@gmail.com (A.A.R.K.); 4 National Institute for Medical Research, Tanga Center, Tanga, P.O. Box 5004, Tanzania; b.mmbando@yahoo.com (B.P.M.); sethmdj2002@yahoo.com (M.D.S); hwmakunde@hotmail.com (W.H.M.); jpalusingu@gmail.com (J.P.A.L.); 5 Neglected Tropical Diseases Control Programme, Dar es Salaam 40478, P.O. Box 743, Tanzania; maligana@gmail.com; 6 Pharmacovigilance and Clinical Trials, Botswana Medicines Regulatory Authority, Gaborone 999106, Botswana; partha18@gmail.com

**Keywords:** circulatingfilarial antigen, microfilaremia, antigenemia, mass drug administration, ivermectin, albendazole, lymphatic filariasis, microfilariae, *Wuchereria bancrofti*, Tanga

## Abstract

Lymphatic filariasis (LF) is a neglected tropical disease targeted for elimination as public health problem through morbidity management and preventive annual mass drug administration (MDA). This cross-sectional community-based surveillance assessed the prevalence and correlates of LF infection in Mkinga district, Tanga-region, Tanzania. A total of 4115 individuals (49.7% males, 35.2% children) were screened for circulating filarial antigens (CFA), microfilaremia (mf) and disease manifestations in 15 villages between November 2018 and January 2019. MDA uptake in the previous year was assessed. Overall prevalence of CFA-positivity was 5.8% (239/4115; 95% CI: 5.1–6.6), with significant heterogeneity between villages (range 1.2% to 13.5%). CFA-positivity was higher in males (8.8%) than females (3.3%), and correlated with increasing age (*p* < 0.001). Prevalence of mf among CFA-positives was 5.2%. Only 60% of eligible inhabitants in the study area took MDA in the previous year, and CFA-positivity was 2-fold higher in those who missed MDA (*p* < 0.0001). Prevalence of scrotal enlargement, hydrocele, arms or legs swelling, lymphoedema and lymphadenopathy was 6.4%, 3.7%, 1.35%, 1.2% and 0.32%, respectively. Compared to baseline data, 16 years of MDA intervention significantly reduced LF transmission and morbidity, although the intended elimination target of <1% mf and <2% antigenemia to level where recrudescence is unlikely to occur by the year 2020 may not be attained. The finding of hotspots with ongoing transmission calls for intensified control measures.

## 1. Introduction

Lymphatic filariasis (LF) is a painful and disfiguring neglected tropical disease (NTDs) that is preventable. The disease is endemic in Sub Saharan Africa (SSA) and is caused by the filarial nematode worm *Wuchereria bancrofti*. The World Health Organization (WHO) baseline data in the year 2000 indicated that more than 120 million people were infected globally, and approximately 40 million suffered from the stigmatizing and disabling clinical manifestations of the disease, including 15 million who have lymphoedema and 25 million men who have urogenital swelling, principally scrotal hydrocele [1]. In 2000, about 40% of LF infected people were from SSA, with cases ranging from 46 to 51 million, and an estimated at-risk population of 432 million people [2]. In 2018, the total estimated at-risk population requiring intervention in Africa was 341 million [3]. LF is a leading cause of long-term disability worldwide causing lymphoedema (elephantiasis), hydrocele, and adenolymphangitis [4,5]. 

The life cycle of *W. bancrofti* involves two hosts—humans and mosquitoes. The adult parasites reside in the lymphatics of the human host. After mating, the first stage larvae (microfilariae) are produced and move to the peripheral blood circulation. The mf exhibit diurnal periodicity and are present in deep vein during the day, and migrate to the peripheral circulation during the night. The peak amount of microfilariae (mf) in the peripheral blood attains during a 4-h period (10 p.m. to 4 a.m.), which is an adaptation to the biting behaviors of the vector mosquitoes [6]. Microfilariae are taken up during the night time feeding on an infected host by mosquito species, mainly Culex, Aedes, and Anopheles spp, where it matures into motile larvae and transmitted to a new human host during next feeding. The larvae migrate to the blood stream and move through the lymphatic system to regional lymph nodes, predominantly in the legs and genital areas [7]. The larvae then develop into adult worms over the course of one year and reach sexual maturity in the afferent lymphatic vessels. After mating, the adult female worm can produce thousands of mf that migrate to the peripheral blood stream to repeat the lifecycle.

Early infection (microfilaraemia) is acquired in childhood, because of lack of immunity and high exposure to infective larvae; albeit, the overt chronic disease manifestation is noted later in life [8]. Lymphatic vessels containing various stages of filarial parasite gradually become dilated with non-functional values, impaired contractility leading to abnormal drainage patterns commonly in the extremities and male genitalia, and rarely in female breasts and genital areas [8,9]. 

LF is listed amongst the six eradicable NTDs. In the year 2000, the WHO launched the Global Programme to Eliminate Lymphatic Filariasis (GPELF), with the goal to eliminate LF as a public health problem by 2020. Two strategies were deployed—morbidity management and mass drug administration (MDA) of microfilaricidal drugs—to the entire at-risk population to halt transmission [10,11]. MDA is needed to reduce infection in the community to levels below a threshold at which mosquitoes are unable to continue spreading the parasites from person to person, and new infections are prevented. WHO recommends a pre-transmission assessment survey (pre-TAS) to be conducted after five effective MDA rounds, and the prevalence of infection is less than 1% for mf and 2% for antigenemia. If an implementation unit with >65% coverage passes pre-TAS, then it may progress to a transmission assessment survey (TAS). TAS measures whether evaluation units have lowered the prevalence to a level where recrudescence is unlikely to occur, and transmission is considered unsustainable, even in the absence of MDA [12]. 

In the LF control program, two-drug regimens consisting of a combination of ivermectin and albendazole (IA) or diethylcarbamazine and albendazole (DA) are used during annual MDA to treat the entire at-risk population. Due to safety concerns for diethylcarbamazine, WHO recommends IA in onchocerciasis co-endemic countries [13]. These two-drug regimens usually clear mf that triggers transmission in the community, but with a minimum effect on adult filarial worms [14]. Hence, repeated MDA during the reproductive period and life span of the female adult worms in the human body (estimated to be 6–8 years or more) is required to stop transmission and prevent new infections [15,16]. Some countries now are moving from a two-drug regimen to a three-drug regimen of ivermectin, diethylcarbamazine, and albendazole (IDA) to accelerate progress towards elimination as desired, except in areas endemic for loiasis and onchocerciasis [13,17]. In 2018, 893 million people in 49 countries were living in areas that require preventive chemotherapy to stop the spread of infection [3].

Tanzania ranks as the third African nation in terms of the highest-burden of LF, with over 34 million people at risk and more than 6 million infected [18,19,20]. The risk of being infected is particularly high in the coastal areas along the Indian Ocean, including Tanga region, albeit infections are reported in other mainland areas [20,21,22]. The Tanzanian National Program for Elimination of Lymphatic Filariasis (NPELF) started MDA consisting of IA in most parts of the country, including Tanga region in 2002. 

Previously, few communities and school-based surveillance studies assessed the short and mid-term impact of MDA in different districts of Tanga region after three [19], six [23], and eight [24] rounds of MDA. Human microfilariae initially decreased rapidly following this intervention, leading to a considerable reduction in transmission, but the effects thereafter leveled off, and transmission continued at a low level after the third MDA [19]. Before the MDA intervention, the prevalence of mf and CFA in the community was 24.5% and 53.3% respectively, which considerably decreased after six rounds of MDA to 2.7% and 19.6%, respectively [23]. A cross-sectional spot-check survey after eight rounds of MDA reported a respective prevalence of 15.5% and 3.5% for CFA and mf, respectively, in three communities (a decrease of 75.5% and 89.6% from baseline, respectively) and 2.3% for CFA in school children (decrease of 90.9% from baseline) [25]. In some endemic areas, a reduction of infection prevalence to low levels as 1% was noted, but along the coast of the Indian ocean after eight rounds of MDA, the prevalence of infection was still higher than what was expected [24,26]. In line with this, transmission by the three vectors (*Anopheles gambiae*, *An. funestus* and *Culex quinquefasciatus*) as determined by dissection declined sharply with overall vector infectivity rate of 99.3% and mean monthly transmission potential of 99.2% between pre-interventional and fifth post-interventional period. A major shift in vector species composition, from predominantly anopheline to almost exclusively culicine was observed [23]. 

Monitoring effective drug coverage and continued surveillance after a decade of program implementation is important to measure the impact of long-term MDA in reducing the burden of LF in endemic countries. This is important, particularly as the GPELF target is to eliminate LF as a public health problem by the year 2020, and surveillance data is critical for evidence-based decision making. In this study, we report the current prevalence of LF infection and clinical manifestations after sixteen rounds of MDA and uptake of MDA in the previous year in a highly LF endemic region of Tanzania.

## 2. Experimental Section

### 2.1. Ethical Statement

Ethical clearance was granted by the Medical Research Coordinating Committee (MRCC) of the National Institute for Medical Research, Tanzania (Certificate No. NIMR/HQ/R.8a/Vol. IX/2890). Prior to the study, separate meetings with district authorities and village leaders were conducted to obtain permission to conduct research in the community. Sensitization meetings were held in each village to explain the objectives and methodology of the study, as well as to seek community consent. Questions were asked, and explanations provided, by researchers during these meetings. Informed consent was obtained in writing from all individual participants and/or parents/guardians for children during the survey. Confidentiality of participants’ information was maintained during and after the study.

### 2.2. Study Design and Area 

This was a community-based cross-sectional study that aimed at screening for both CFA and mf to determine the prevalence of LF infection and disease in a highly endemic region. The study was conducted between November 2018 and January 2019. The Tanga region, located along the coast of Indian Ocean, has 8 districts—Handeni, Kilindi, Korogwe, Lushoto, Mkinga, Muheza, Pangani, and Tanga. This study was conducted in the Mkinga district. The district is bordered by Muheza district and Tanga city to the South, Indian Ocean to the East, Korogwe and Lushoto districts to the West and Kenya border to the North. The district has two main rainy seasons in a year (bimodal): the long rains from March to June, and the less intensive short rains from November to December. Tanga is a warm and wet climate region, with no significant variation of temperature at the coast, due to the influence of the Indian Ocean. Humidity is high, and often goes up to 100% maximum and ranges from 65% to 70% minimum. There are health facilities in most villages in the district, and the majority of the population has access to a health facility within a distance of about 6 km. According to the national census conducted in 2012, the Mkinga district population was 118,065, with 48.9% (57,760) being male, and the population density was 44 people per sq.km. The current population (2019) is projected to be around 250,000, and the altitude ranges from 0 to 1506 m above sea level, measuring from the Nilo peak [27]. The main economic activities in this district include fishing, subsistence farming, low-scale livestock keeping, and petty trading for the rest. 

**Figure 1:**
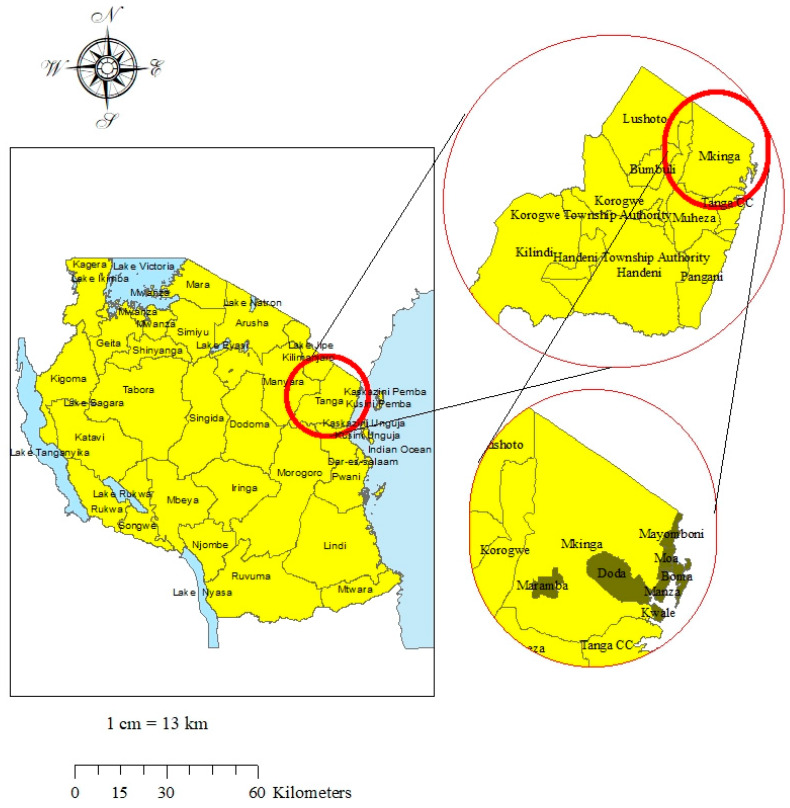
Map of the study site. Left is the map of Tanzania located in the Eastern part of Africa. The top-right figure shows the map of Tanga region, where the Mkinga district is located. The bottom-right figure shows the map of wards in the Mkinga district, whereby villages in these wards participated in this study. The study site map was originally generated using ArcGIS software version 10.7.1 [28].

The NLFEP in the Mkinga district was implemented since 2002. By 2018, the program had administered 16 rounds of annual MDA. A map indicating the location of the study sites in the Tanga region is depicted in Figure 1.

### 2.3. Selection of Study Villages and Study Population

Purposive sampling technique was used to select 15 villages based on the past transmission trends, as assessed by CFA [25]. This was essentially a convenient technique to recruit participants with clinical conditions and laboratory results consistent with study objectives. The sampled villages were backed up with a review of epidemiological data from the Neglected Tropical Diseases Control Programme (NTDCP). Study villages included: Kichalikani, Kizingani, Kwale, Mongavyeru, Mwandusi, Tawalani, Manza, Kichakamiba, Subutuni, Zingibari, Moa, Ndumbani, Mayomboni, Bamba-Mwarongo (B’Mwarongo), and Maramba A. Most of the selected villages are located along the coast of the Indian Ocean, except for B’Mwarongo and Maramba A, which are located further inland. Initial meetings with district and village authorities were conducted explaining the aim, objectives, benefits, and model for recruiting study participants living in the study area. This was followed by community sensitization meetings, which were conducted in each village to inform the community about the purpose, methodology, and significance of the proposed research, and to obtain community consent. Health facilities (dispensaries), village administrative buildings, or schools were selected as meeting points for the screening exercise. One day before the date scheduled for screening, communities were re-sensitized by the help of Hamlet criers, who used horn speakers or house-to-house visits to deliver information.

A sample size of 4000 was considered sufficient to estimate the community prevalence of CFA of 5%, with a precision of 20%, alpha of 0.05, and a power of 80%. Convenience sampling method was used during the screening for CFA testing where all participants were asked to attend a designated study center in each village after the sensitization meeting with the community through their community leaders.

### 2.4. Clinical and Parasitological Examination 

All participants eligible to take part in MDA (aged five years or above) were enrolled, as MDA medicines are only given in this age group. Enrolled participants were registered and provided with a special identification card with personal details and a unique barcode. At the registration station, a questionnaire was used to collect socio-demographic information of participants, including age, sex, duration of residence in the village, and personal contacts. Biometric measurements to determine height and weight were taken using a height measuring bar and weighing scale, respectively.

A semi-structured questionnaire was used to collect clinical and recent drug intake history, as well as other symptoms associated with LF morbidities, such as the history of use of MDA and the presence of lymphoedema and hydrocele. A general clinical examination was performed to all participants with or without symptoms or signs. Physical examination was conducted in all individuals aged 5 years and above to detect lymphoedema of the lower and upper limbs in both males and females, whereas, in males, the scrotal swelling (hydrocele) was examined as described previously [29]. Females were assessed for swollen lymph nodes. Clinical examinations were performed by four (4) trained medical doctors, who were also part of the study team and had adequate experience on treatment and management of LF. Apart from the Doctor of Medicine degree and further postgraduate trainings, the doctors had over 10 years’ experience in parasitological work at the coast region. The diagnoses were therefore made from clinical presentation of study participants. 

All participants were screened for CFA using Alere^TM^ Filariasis Test Strip (FTS) (Alere©, Waltham, United States), following the manufacturer’s instructions. In brief, after cleaning with an alcohol swab, a finger prick was made using a sterile disposable lancet to collect blood sample. Approximately 75 µL of blood was applied on the FTS using a special pipette with a 75 µL mark and results were read in 10 min. FTS detects CFA released in the blood by adult worms. FTS positive tests were read by two researchers and positives retested. Conversely, to ensure quality control, the readings for mf for each night blood sample was done by two well trained and experienced laboratory technicians, who discussed and agreed on the results. All positive tests were also reviewed by the study leader or designated scientist.

### 2.5. Detection for Microfilaraemia

All antigenemia positive participants were eligible for night blood sampling for the determination of mf. Blood sampling was conducted at a village meeting point (health facility or village office building) in most cases. A door-to-door approach was also used under special circumstances, especially when participants did not show-up for blood sampling. Finger prick blood samples were taken from the study participants in the night between 21.00 and 01.00 h onto 75 μL capillary tubes. Each blood sample was dispensed into a 1.8 mL cryo-tube containing 900 µL of 3% acetic acid, mixed thoroughly and transferred into Sedgwick-Rafter counting chamber. LF parasitaemia was estimated by counting the number of microfilariae in the chamber, using a compound light microscope set at 4× magnification located at the National Institute for Medical Research (NIMR) laboratory in Tanga. Results were reported as number of microfilariae per 75 µL of blood. Double reading of rafter membrane was done for quality assurance of the data.

### 2.6. Data Management and Analysis

Data was collected electronically using tablets and submitted daily to the central server at the NIMR laboratory in Tanga. The open source data kit (ODK, https://opendatakit.org/) software was used to create the database and data collection applications. The data manager reviewed the collected data daily for precision and consistency and queries generated were sent back to the head of each study team for resolution. Data cleaning and validation was done continuously, and periodic reports generated. Final cleaning was done after the survey, and analysis was performed according to the data analysis plan.

Data were analyzed using STATA version 13 and *R* statistical software [30] Descriptive statistics was used to calculate the proportion of study participants that were CFA and mf positive, and the mean mf parasite intensity. Continuous variables with normal distribution were compared using *t*-test or ANOVA test. Skewed continuous variables were presented as median with interquartile ranges (IQR), and were compared using sign rank tests, while categorical data were compared using Chi-square test.

Chi-square test was used to determine whether age was associated with CFA and scrotal enlargement. Binary logistic regression analyses were performed to generate odds ratios (OR) including 95% confidence intervals (95% CI) to assess potential relationships between age, sex, missing MDA, and living in hotspots (exposures), and CFA positivity (outcome). Significance level was set at 0.05 and the confidence interval at 95%. All *p*-values were two-sided and a *p* value of less than or equal to 0.05 was considered to indicate statistical significance.

## 3. Results

A total of 4115 individuals from 15 selected villages in the Mkinga district, Tanga region were screened for CFA, of which 2045 were males (49.7%). There was a variation in age of the study participants, with Ndumbani village having the oldest population (median age 32 years), whereas Mayomboni and Zingibari had the youngest (14 years). The median age of participants was 22.7 years (IQR = 12.5–44.5 years), being significantly higher among females (median = 25.6, IQR = 12.8–45.0) than males (median = 20.0 years, IQR = 12.2–43.9), *p* = 0.008. Out of 4115 individuals screened, 1447 (35.2%) were children aged 5–14 years, Table 1.

### 3.1. Prevalence and Correlates of Circulating Filarial Antigen 

**Figure 2:**
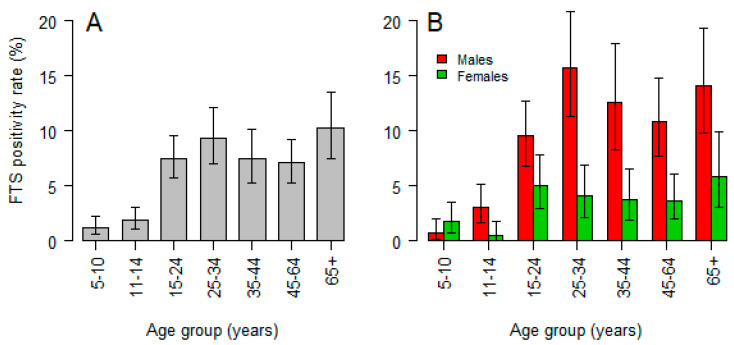
Prevalence and 95% CI of circulating filarial antigens (CFA) positivity stratified by age group (**A**) and by age group and sex (**B**).

The overall prevalence of CFA positivity was 5.8% (239/4115), 95% CI: 5.1–6.6), with males having significantly higher rates (8.3%) compared to females (3.3%), *p* < 0.001. Analysis further shows that there was a positive trend of increase of the prevalence of CFA positivity with increasing age (*χ*^2^ = 53.83, *p* < 0.001), with a maximum of 10.2% being among age group of 65+ years. However, there was a spike (9.3%) among 25–34 years old age group (Figure 2A). The pattern of LF infection by age was similar between females and males. However, after adjusting for the effect of age, the risk (odds ratio) for being positive in males was 2.9-fold higher (95% CI: 2.14–3.85, *p* < 0.001) when compared to females, Figure 2B. 

The prevalence of antigenemia varied significantly between villages ranging from 1.2% to 13.5% (Table 2). Analysis by ward and village levels showed that individuals living at Mongavyeru village in Kwale ward were the most affected. The overall prevalence in this village was 13.5% (95% CI: 10.8–16.2). Similarly, high prevalence was observed at Kizingani (8.9%), Maramba A (8.4%), and Ndumbani (7.6%). The prevalence in children below the age of 5 years was highest in Kwale village (7.7%), followed by those from Kizingani and Mongavyeru, with 3.6% each (Table 2). Generally, the population in Kwale ward was the most infected.

Binary logistic regression analysis indicated that being older age, male, missing last MDA, never taking ivermectin and living in Kwale, Maramba or Moa wards were significant correlates of CFA positivity (Table 3). Maramba A was identified as a hotspot for LF in Mkinga district. 

### 3.2. Prevalence and Correlates of Microfilaremia

Out of 239 individuals who tested positive for CFA, 28 (11.7%) were not available for the night blood specimen collection for the detection of mf using microscopy. Eleven individuals out of 211 were mf positive, giving a prevalence of 5.2% among positive CFA individuals. Microfilaraemia was detected in four out of 15 villages—Kizingani (4/35, 11.43%), Mongavyeru (5/71, 7.0%), and one from each of B’Mwarongo (1/7, 14.3%) and Maramba A (1/18, 5.6%). The youngest mf positive child was 11 years old from Kizingani village. From the observed prevalence, mf among individuals tested for CFA assuming a 100% sensitivity of FTS test, the overall prevalence of positive microfilaria among the screened study participants was estimated to be 0.30%.

The mean parasite intensity was estimated to be 9.8 (95% CI: 4.04–22.2) parasites per 100 μL of blood. The youngest child from Kizingani village who was positive for mf had 32 parasites per 100 μL of blood, while the mean parasite intensity in Mongavyeru village that had the majority of individuals positive for mf (five individuals) was 14.0 parasites per 100 μL of blood.

### 3.3. Coverage of MDA from the Previous Round

**Figure 3:**
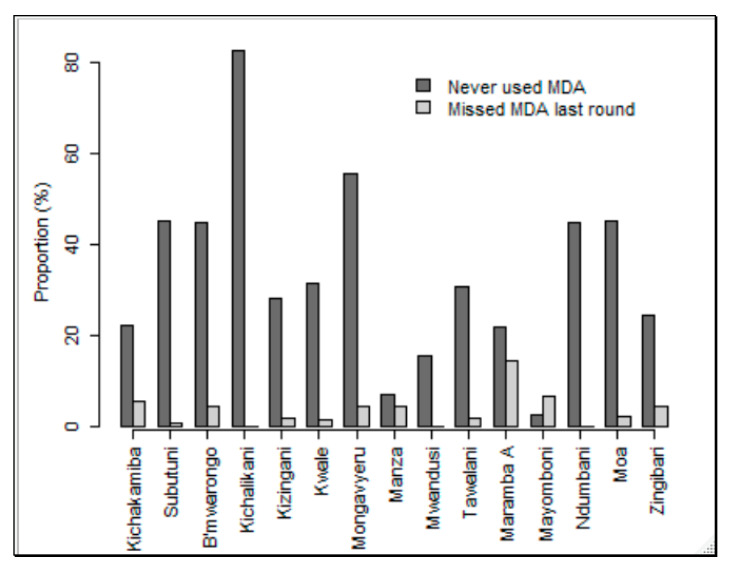
Proportion compliance of villages with mass drug administration (MDA) coverage.

Coverage of MDA during the last round of distribution (2017) was explored in 3932 individuals from the age of 6 years, of which 1553 (39.5%) indicated not to have taken the medicines. The proportion of individuals who did not take drugs was slightly higher in males (40.9%) than in females (37.9%), *p* = 0.06. Similarly, a question of whether a person had ever used ivermectin was assessed for the same population, of which, out of 3846 interviewees, 1339 (34.8%) responded they had never used the drug, with a slightly higher frequency in males (36.2%) than in females (33.4%). The prevalence of CFA positive individuals who did not take MDA drugs in the previous year (8.25%, 134/1625) was significantly higher than those who took (4.18%, 100/2392) (*χ*^2^ = 29.15, *p* < 0.0001, odds ratio = 2.056; 95% CI of OR = 1.5771 to 2.6905). Results further suggest that there was systematic non-compliance in the study villages, with Kichalikani village having the most MDA non-compliant participants in 2017. There was a slightly higher proportion than the average of individuals who were compliant but missed MDA during the last round in Maramba A village, as shown in Figure 3.

**Figure 4:**
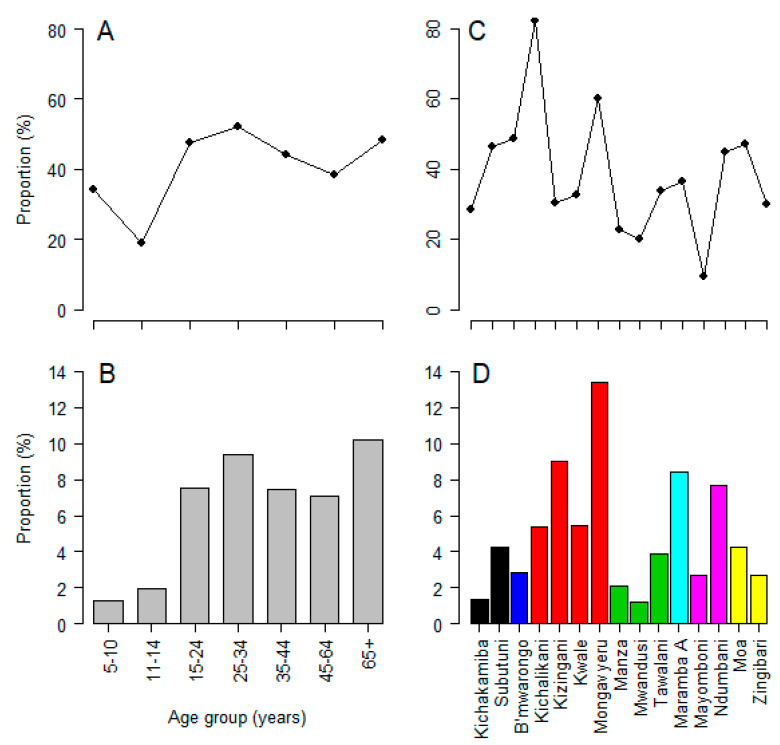
Proportion of individuals who did not use MDA in the last round of distribution by age group (**A**) and by villages (**C**); and prevalence of CFA by age group (**B**) and by village of residence (**D**). The color bands represent Boma, Doda, Kwale, Manza, Maramba, and Mayomboni wards.

Analysis across the age groups indicated that the proportion of individuals who did not take MDA during the last round (previous year) or never used ivermectin before was lower in children aged 11–15 years (18.8%) than adults. There were significant differences in the proportion of individuals who missed MDA in the previous year amongst the various age groups, the highest being in the 25–34 years age group (52.3%) and 65+ (52.3%) *p* < 0.0001. The trend of use of MDA in the previous year (in 2017) by age group mirrored precisely that of CFA positivity, except for the youngest age group (Figure 4A). The same pattern of high CFA positivity was also observed in villages with a history of low MDA compliance during the last round (Figure 4).

Data on MDA overage for the Mkinga district between 2004 and 2018, as obtained from NTDCP, is summarized in Table 4:

### 3.4. Clinical Manifestation of Bancroftian Filariasis

**Figure 5:**
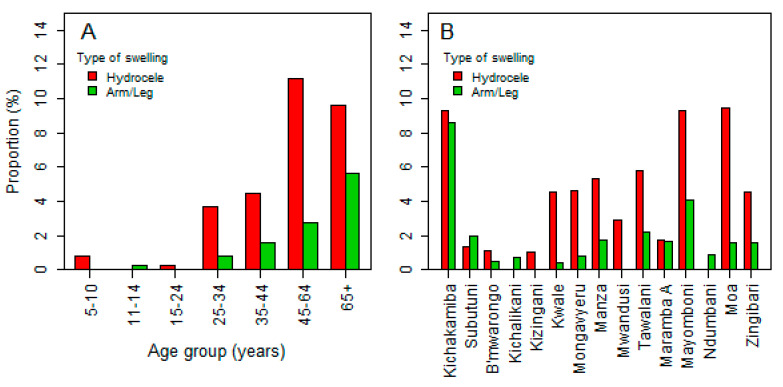
Distribution of hydrocele in males and swelling of arms or legs in both males and females by age group (**A**) and by village (**B**).

Data on scrotal enlargement was obtained for 1988 males, of which 128 (6.4%) had an enlarged scrotum, and the rate of scrotal enlargement was increasing with age (*χ^2^_trend_* = 126.3, *p* < 0.001). Scrotum enlargement was significantly higher among males with positive CFA (14.8%), compared to those with a negative test (7.9%), *p* = 0.006. Hydrocele was found in 73/1992 (3.7%) males, which is 57.5% (73/127) of those with an enlarged scrotum. However, hydrocele prevalence was not significantly different between those with positive CFA tests (4.8%) against those with a negative test (3.6%), *p* = 0.42. Swelling of arms or legs was observed at a lower frequency of 55/4074 (1.35%), and these were mainly in individuals with negative CFA tests (53 cases), albeit there was a similar pattern with other types of swellings (Figure 5A). It was interesting to note a variation of prevalence of swelling with the village of residence, although the pattern did not mimic the CFA positivity tests, as villages with the highest CFA prevalence were observed to have a lower prevalence of swellings (Figure 5).

Lymphoedema and lymphadenopathy were also observed at a low frequency. The prevalence of lymphoedema was 1.2% (50/4059), while lymphadenopathy 0.32% (13/4065). In both types of edema, the youngest child with the condition was aged 13 years, while the median age was 60.5 years (IQR: 44–70) for lymphoedema and 53 years (IQR: 44–65.6) for lymphadenopathy, and both were not associated with positive CFA tests, *p* > 0.5. 

## 4. Discussion

We conducted a cross-sectional community-based surveillance to identify the current status of LF infection and disease manifestations in the Mkinga district, a highly endemic rural area in north-eastern Tanzania. Main findings from this study include that the prevalence of antigenemia positivity being 5.8%, with significant heterogeneity in infection rate between villages (ranging from 1.3% to 13.5% in the communities, and 0% to 7.7% amongst children). The prevalence of microfilaraemia amongst those who tested positive for antigenemia was 5.2%. The prevalence of antigenemia was significantly higher in males (8.3%) than females (3.3%), and positively correlated with increasing age. Missing MDA in the previous year was significantly associated with a high risk of CFA-positivity. We identified two at-risk age-groups (25–34 years and 65+ years old) who mostly missed MDA in the previous year and had the highest infection rate. These age groups may serve as reservoirs of infection for continued transmission, and hence the need for targeted intensive control measures. Our findings, based on screening of 4115 individuals from 15 selected villages, reflect the current status of LF infection and clinical manifestation in the Mkinga district, Tanga region. To our knowledge, this is the largest sample size surveillance study evaluating the impact of long-term MDA in reducing infection transmission and disease morbidity after decades of program implementation from the LF endemic region in sub-Saharan Africa.

In the Mkinga district, annual MDA containing IA was initiated in 2002. According to the NTDCP mapping data, the prevalence of LF in Mkinga district was 62.0% in 2002, 3.70% in 2014, and 6.0% in 2017. The NTDCP report is based on prevalence data obtained from hotspots only, which are not more than two villages in one district [32]. The prevalence data obtained from the program is inadequate to show with certain any trend as mapping was done at long intervals. The CFA-positivity in Mkinga district (6%) reported by NTCDP in 2017 is comparable to the observed overall antigenemia (5.8%) a year later in this study. This may indicate a waning effect of MDA, although the infection rate varied greatly between villages. Maramba A and Maramba B were the two selected villages for the 2017 NTDCP survey. The 8.4% prevalence of CFA positivity in Maramba A (Table 2) is higher than the overall 6.0% prevalence for Maramba A and B reported by NTDCP in the previous year. Nevertheless, it is obvious that compared to the baseline data in 2000, long-term MDA intervention has significantly contributed to reducing the burden of LF and reduced transmission in the community. However, our data indicates the intended threshold of mf (<1%) and CFA (<2%) to a level where recrudescence is unlikely to occur have not been achieved in most of the studied villages. 

A wide range of CFA positivity across the 15 study villages was observed, the lowest being 1.2% in Mwandusi village (Manza ward) and highest being 13.5% in Mongavyeru village (Kwale ward). Based on CFA results, prevalence is still high in most of the study villages, since only two villages (Mwandusi, 1.2% and Kichakamiba, 1.3%) were found to have CFA levels of <2% (Table 2). On the other hand, Mongavyeru (13.5%) and Kizingani (8.9%) villages, in the Kwale ward, and Maramba village (8.4%) had the highest prevalence in the community. The prevalence amongst children in these villages was also high indicating clustering of infection and hotspots with ongoing transmission. Congruent to our finding, the existence of ‘hotspots’ and spatial clustering of infections after eight rounds of MDA was also reported in India [33]. Our results indicate the need for strengthening surveillance strategies for detecting new hotspots, and careful selection of sentinel and spot-check sites for TAS. Pre-TAS is recommended after at least five rounds of MDA, each with >65% coverage and with mf-prevalence of <1% in both sentinel and spot-check sites [12].

CFA positivity exhibited heterogeneity with some villages and wards having more positive individuals. This could be a result of varying exposure to infection as a result of the vicinity to breeding sites, the presence of infected individuals who act as sources (reservoirs) of infection, as well as MDA coverage. In this study, Mongavyeru village in Kwale ward had the highest prevalence of CFA and is also amongst the villages with a poor history of compliance to MDA (Figure 4). Some villages depicted low prevalence of CFA, which might be due to various interventions creating synergy besides the MDA, such as the use of insecticide-treated nets or local insecticide repellants (smoke, herbs), as observed in studies conducted elsewhere [34]. In some cases, the presence of climatic and environmental conditions that are unfavorable for vector survival could contribute to low infection or positivity rates, even in areas where compliance to MDA is low [35]. However, this study did not explore the entomological characteristics and, therefore, no link could be established in this case, albeit other studies had reported similar conclusions [36].

The prevalence of microfilariae in CFA positive individuals was 5.2%, and when computed against all screened individuals, it was less than 1%. This is a good sign to the LF control efforts, since less mf in blood implies a potential reduction in transmission. Most studied villages were negative for mf. Although the overall prevalence of mf was at 0.3%, which is below cut-off point(<1%) in areas where Culex and Anopheles species are principal vectors [37,38], the prevalence in Kizingani village was found to be 1.2%, which is above the threshold. In comparison, it was 0.8% in Mongavyeru village. Importantly, the fact that mf was observed in young individuals up to 11 years in one of the study villages, this potentially signifies ongoing transmission in this area. This highlights the need to adopt stratification in planning for interventions, since there is spatial heterogeneity in LF prevalence across different geographical areas, as observed in previous studies [26,39,40].

Antigen-positive individuals were identified in all age groups (Figure 2). The increasing trend of CFA positivity with age signifies the role of adult parasites as they mature in infected individuals, and this could be a sign of ongoing transmission. The FTS detects CFA released in the blood by adult worms [41], who produce mf through mating. IA kills the mf but has little effect on adult worms, hence the need for repeated MDA during the life span of the adult worm [15,16]. MDA with IA halts transmission of mf from an infected person to another, by reducing the number of mf in the blood to a level whereby recrudescence is unlikely to occur. In areas with missed MDA, the transmission of mf will be high and hence high levels of CFA prevalence. The prevalence of CFA-positivity was more pronounced amongst individuals aged 15–34 years and beyond 65 years. The proportion of individuals who missed MDA in the previous years was also high in these two age groups, indicating that non-compliance to MDA correlates with the observed high infection rates in these age groups. 

The observed high prevalence of antigenemia in males as compared to females is in line with results from other studies [19,21,23,40]. Men are exposed to more risk of infectious mosquito bites, as they are engaged in fishing and stay outdoors until late hours, coinciding with the peak hours for mf circulation in peripheral blood and, thus, more transmission propensity. As observed in this study, males had a high tendency of missing MDA, due to their constant absence from their homes during drug distribution rounds, as well as refusal to take drugs as a result of misconceptions of the adverse events of the drugs used for MDA [42,43,44]. In terms of accessing health facilities, even at the community level, women are more concerned about their health status than men [45]. In this study, those who turned-up for medication were mostly women. 

According to the GPELF progress report of 2017, Tanzania is listed amongst countries that have scaled-up MDA to all endemic districts [46]. In this study, about 40% of the study participants did not take drugs for MDA in the previous year (in 2017). The proportion of individuals who did not take IA in the last round was observed to be higher in males than females. In our study, the coverage, as reported by interviewed individuals, was 60%, which is lower than the reported coverage data obtained from the program for the Mkinga district (78.5%) or the national MDA coverage (84.2%) in 2017, or the WHO recommended effective coverage of >65% of the targeted population [46]. However, program data is based on assessment at hotspots only but not in many other villages, which also have varied population sizes and different habits in adhering to treatments. Interestingly, compared to those who received MDA the previous year, the CFA positivity rate was two-fold higher in those who did not receive MDA. Low MDA coverage reduces the impact of MDA on transmission and delay in reaching the elimination targets [46]. Thus, low MDA coverage could be one of the contributing factors for not reaching the LF elimination target in Tanzania. 

In this study, there were reduced scrotal swelling (hydrocele) and leg/arm swelling (lymphoedema) rates in the young age group (11–24 years) compared to the older group (≥25–65 years). This indicates that the reduction of new infections resulted in lowered progression of infection to chronic pathologies, compared to the findings of previous studies in the area [19,29]. Small scrotal swellings in the age group of 5–11 years could be a sign of early stages of hydrocele (sub-clinical hydrocele). This could be triggered by the host human-immune and parasite interaction, leading to the clearance of acute infection, which could have led to chronic clinical disease (hydrocele and lymphoedema) observed. In Figure 5A, the scrotal enlargements in the younger age group (5–11 years) were not hydrocele, but rather a condition possibly triggered by other conditions, such as congenital inguinal hernias, which are frequently seen in this age group [47], tumors, or tuberculosis. In this age group, hydroceles were not as common as in individuals aged 16–24 years [24,29]. The frequencies of hydrocele observed in this study were similar to those reported in other studies done previously in the study area and elsewhere [18,24]. High proportions of scrotal swelling, which were not hydrocele, were observed in six study sites (Kichakamiba, Mongavyeru, Tawalani, Maramba A, Mayomboni, and Moa). A similar trend of arm/leg swellings was also observed in the study sites. Ndumbani village was the only study site where scrotal swelling was not observed, although CFA was amongst the highest (7.6%).

Our finding highlights the need to assess transmission dynamics in the areas with high infection rates focusing on the MDA implementation strategies (advocacy, compliance to drug intake, and training of drug distributors). Reasons as to why community members are not taking MDA to warrant successful elimination also needs to be explored through an anthropological study. The NTDCP should continuously monitor endemicity levels and MDA coverage for LF. Villagers in the district still experience clinical manifestations, and, therefore, preventive chemotherapy and management of morbidity (lymphoedema and hydrocele) need to be sustained. The WHO should also consider reviewing its GPELF targets and probably adjust the goals for the elimination of LF.

## 5. Conclusions

Overall, the prevalence of mf in most villages was <1%, but only two out of 15 villages attained the intended prevalence antigenemia level of <2% cut-off point. Since the levels are still high, pre-TAS and subsequent TAS cannot be initiated at this time. Albeit, the infection rate was generally low in most villages, and the high prevalence of CFA-positivity in some villages and the detection of mf in children is a concern. The finding of infection hotspots with signs of ongoing transmission, partly due to non-adherence to MDA, calls for targeted intensive control measures. The high-risk group for mosquito exposure in this case 25–34 years and 65+ old, should be considered for targeted interventions.

The overall prevalence of LF was comparable to last year (2017), with few sites showing signs of reduced CFA positivity. There was low MDA coverage (60%) in this district as compared to figures recommended by WHO (i.e., 65% of an implementation unit). Nevertheless, compared to the baseline data and over 16 rounds of MDA, the intervention in the community significantly reduced the disease burden and morbidity in Mkinga district. However, considering the current findings, the GPELF target for elimination of LF as a public health problem by 2020 may not be attained at least in the studied areas.

## Figures and Tables

**Table 1:** Demographic characteristics of study participants.

Ward Name	Village Name	All Participants			Children (5–14 Years)	
		*N*	Mean Age (Years)	Median Age (IQR)	*N*	Median Age (IQR)
Boma	Kichakamiba	151	32.1	17.8 (14.0–46.3)	43	12.8 (11.3–13.7)
	Subutuni	257	35.2	31.4 (16.5–50.0)	54	9.9 (7.4–12.0)
Doda	B’Mwarongo	216	29.8	16.3 (11.2–47.0)	105	11 (10.0–13.0)
Kwale	Kichalikani	262	33.3	29.0 (15.4–47.4)	63	10 (6.8–11.8)
	Kizingani	403	29.4	20.0 (11.0–44.0)	149	9.5 (7.0–12.0)
	Kwale	225	30.4	27.5 (13.0–41.6)	70	9.9 (7.0–13.0)
	Mongavyeru	600	26.8	22.0 (12.0–40.0)	203	9.4 (7.0–12.0)
Manza	Manza	291	26.1	16.4 (13.3–36.4)	108	13.2 (12.2–13.7)
	Mwandusi	332	27.1	23.0 (11.2–38.7)	128	9.9 (7.8–12.2)
	Tawalani	181	39.1	37.0 (20.0–55.0)	29	9.2 (7.0–12.0)
Maramba	Maramba A	238	31.3	26.8 (12.0–46.2)	81	10 (7.0–12.0)
Mayomboni	Mayomboni	74	23.3	13.7 (13.0–29.0)	51	13 (12.4–13.9)
	Ndumbani	223	34.7	31.6 (15.4–50.0)	53	9.4 (7.4–11.4)
Moa	Moa	212	33.3	29.0 (13.0–49.0)	68	10.3 (7.9–12.8)
	Zingibari	450	25.5	14.0 (11.0–36.1)	242	11 (9.0–13.0)
Total		4115	29.9	22.7 (12.5–44.5)	1447	11 (8.0–13.0)

**Table 2:** Distribution of CFA positivity rate by village’s general study population and in children aged 5–14 years.

Ward Name	Village Name	General Population			Children (5–14 years)		
		*N*	% Positive	95% CI	*N*	% Positive	95% CI
Boma	Kichakamiba	151	1.3	0.1–4.7	56	0	0–6.4
	Subutuni	257	4.2	2.1–7.5	57	1.8	0–9.4
Doda	B’mwarongo	216	2.8	1.02–5.9	107	1.9	0.2–6.5
Kwale	Kichalikani	262	5.3	2.9–8.8	68	1.5	0–7.9
	Kizingani	403	8.9	6.3–12.0	165	3.6	1.3–7.7
	Kwale	225	5.3	2.7–9.1	78	7.7	2.9–16.0
	Mongavyeru	600	13.5	10–16.4	221	3.6	1.6–7.0
Manza	Manza	291	2.1	0.7–4.4	134	0.7	0–4.1
	Mwandusi	332	1.2	0.3–3.1	139	0.7	0.01–3.9
	Tawalani	181	3.9	1.5–7.8	34	0	0–10.0
Maramba	Maramba A	238	8.4	5.2–12.6	91	2.2	0.3–7.7
Mayomboni	Mayomboni	74	2.7	0.3–9.4	54	0	0–6.6
	Ndumbani	223	7.6	4.5–11.0	58	0	0–6.2
Moa	Moa	212	4.2	1.9–7.9	70	0	0–5.1
	Zingibari	450	2.7	1.3–4.6	250	0	0–1.4

**Table 3:** Factors associated with CFA positivity using binary logistic regression analysis.

Parameter		B	Std Error	Exp(B)	95% CI for Exp(B)	*p*-Value
Age		0.019	0.003	1.019	1.014–1.025	<0.0001
Sex (ref female)		0.970	0.147	2.637	1.977–3.518	<0.0001
Bed net use		−0.059	0.218	0.943	0.615–1.446	0.78
House windows screen		−0.216	0.145	0.805	0.607–1.070	0.14
Use of indoor spray		0.232	0.245	1.261	0.780–2.039	0.34
Missed last MDA		0.723	0.136	2.060	1.577–2.691	<0.0001
Never used Albendazole		0.820	0.144	2.271	1.712–3.013	<0.0001
Never used Ivermectin		0.791	0.137	2.205	1.686–2.885	<0.0001
Ward	Boma	0.005	0.359	1.005	0.497–2.029	0.99
	Doda	−0.137	0.470	0.872	0.347–2.190	0.77
	Kwale	1.176	0.239	3.240	2.030–5.172	<0.0001
	Manza	−0.417	0.331	0.659	0.345–1.260	0.21
	Maramba	1.030	0.322	2.800	1.489–5.265	0.001
	Moa	0.735	0.325	2.086	1.104–3.942	0.024

**Table 4:** MDA coverage in Mkinga District—2004–2018. Source: [31].

Year of MDA	Coverage in %
2004	78.4
2006	80.6
2007	76.2
2009	44.6
2010	57.5
2011	52.6
2012	41.0
2013	53.5
2014	72.1
2015	76.0
2016	78.2
2017	78.5
2018	85.0
